# Sphingosine-1-Phosphate Enhances Satellite Cell Activation in Dystrophic Muscles through a S1PR2/STAT3 Signaling Pathway

**DOI:** 10.1371/journal.pone.0037218

**Published:** 2012-05-14

**Authors:** Kenneth C. Loh, Weng-In Leong, Morgan E. Carlson, Babak Oskouian, Ashok Kumar, Henrik Fyrst, Meng Zhang, Richard L. Proia, Eric P. Hoffman, Julie D. Saba

**Affiliations:** 1 Children's Hospital Oakland Research Institute, Oakland, California, United States of America; 2 Genetics of Development and Disease Branch, National Institute of Diabetes and Digestive and Kidney Diseases, National Institutes of Health, Bethesda, Maryland, United States of America; 3 Research Center for Genetic Medicine, Children's National Medical Center, Washington, D.C., United States of America; Blaise Pascal University, France

## Abstract

Sphingosine-1-phosphate (S1P) activates a widely expressed family of G protein-coupled receptors, serves as a muscle trophic factor and activates muscle stem cells called satellite cells (SCs) through unknown mechanisms. Here we show that muscle injury induces dynamic changes in S1P signaling and metabolism *in vivo*. These changes include early and profound induction of the gene encoding the S1P biosynthetic enzyme SphK1, followed by induction of the catabolic enzyme sphingosine phosphate lyase (SPL) 3 days later. These changes correlate with a transient increase in circulating S1P levels after muscle injury. We show a specific requirement for SphK1 to support efficient muscle regeneration and SC proliferation and differentiation. Mdx mice, which serve as a model for muscular dystrophy (MD), were found to be S1P-deficient and exhibited muscle SPL upregulation, suggesting that S1P catabolism is enhanced in dystrophic muscle. Pharmacological SPL inhibition increased muscle S1P levels, improved mdx muscle regeneration and enhanced SC proliferation via S1P receptor 2 (S1PR2)-dependent inhibition of Rac1, thereby activating Signal Transducer and Activator of Transcription 3 (STAT3), a central player in inflammatory signaling. STAT3 activation resulted in p21 and p27 downregulation in a S1PR2-dependent fashion in myoblasts. Our findings suggest that S1P promotes SC progression through the cell cycle by repression of cell cycle inhibitors via S1PR2/STAT3-dependent signaling and that SPL inhibition may provide a therapeutic strategy for MD.

## Introduction

Duchenne muscular dystrophy (DMD) is the most common form of MD, a disease characterized by progressive loss of skeletal muscle strength associated with pathological features including muscle pseudohypertrophy, necrosis and regeneration, variation in fiber size, and eventual muscle replacement by adipose tissue [1]. The disease affects cardiac and skeletal muscles including the diaphragm, and patients usually succumb to the disease in their twenties due to heart and respiratory failure. Mutations in the dystrophin gene are responsible for DMD [2]. The dystrophin protein physically interacts with the dystroglycan-associated complex (DGC), which anchors the sarcolemma internally to the cytoskeleton through dystrophin and externally to the extracellular matrix (ECM), thereby facilitating the distribution of forces generated upon muscle contraction. Disruption of the normal bridge between ECM, DGC and cytoskeleton in patients with DMD reduces membrane stability, resulting in myofiber injury and necrosis [3].

Terminally differentiated skeletal muscle fibers harbor a population of adult stem cells called SCs responsible for the regenerative capacity of muscle [4]. SCs reside beneath the myofiber basal lamina, juxtaposed to the sarcolemma, where they exist in a quiescent state under resting conditions. Disruption of the muscle architecture by traumatic injury or genetic instability results in exposure of SCs to bioactive factors released from injured muscle fiber and its niche, leading to their activation [5]. Activated SCs undergo a burst of proliferation and then migrate from the muscle fiber periphery to sites of sarcolemmal injury. SCs fuse with the injured muscle fiber, thereby generating multinucleated myotubes and stimulating a myogenic program that closely recapitulates development. SCs are fundamental to muscle homeostasis, promoting muscle growth and repair of minor injuries sustained during normal activity [6]. During the early stages of DMD, SC activation and accompanying skeletal muscle regeneration compensate for muscle fiber loss. However, the chronic/degenerative phase of DMD is caused by a failure of regeneration to keep up with ongoing injury and destruction of muscle fibers. This may be accounted for in part by an exhaustion of SC reserves or their myogenic potential. Development of methods that replenish the endogenous SC compartment, allow *ex vivo* expansion of donor SCs for cellular therapy, or enhance the myogenic potential of endogenous or donor SCs are each being explored as therapeutic strategies in DMD [7].

S1P is a bioactive lipid that binds to a family of five G protein coupled receptors [8]. Through activation of S1P receptors (S1PRs) and their G protein partners, S1P modulates the activities of adenylyl cyclase, the Ras/MAP kinase cascade, AKT signaling, phospholipase C and small Rho GTPases, thereby affecting cell survival, proliferation, migration and cell-cell interactions [9]. S1P signaling is essential for many physiological processes including angiogenesis, hematopoietic cell trafficking and development. S1P is generated from sphingosine by a phosphorylation reaction catalyzed by sphingosine kinases (SK), SphK1 and SphK2 [10]. Sphingosine can be regenerated from S1P through the actions of specific and nonspecific lipid phosphatases. However, SPL is responsible for irreversible S1P catabolism and has a major impact on the availability of S1P signaling pools [11]. In addition to its other activities, S1P signaling has been implicated in muscle function, regeneration and the activation and proliferation of SCs in culture [12]–[25]. Rodent muscles have been reported to express three of the five known S1PRs [23]. Importantly, S1P was recently identified as the signal that causes quiescent SCs to re-enter the cell cycle, whereas chemical inhibition of S1P formation prevented muscle regeneration [26]. This suggests a central role for S1P in muscle homeostasis, consistent with our previous finding that *Drosophila* mutants with dysregulated S1P metabolism exhibit a myopathy [27]. However, the mechanism by which S1P activates SCs is not known.

Signal Transducer and Activator of Transcription (STAT) proteins represent a family of transcription factors that play a central role in regulating inflammatory responses [28]. STATs have been implicated in the control of cell proliferation, migration and differentiation. STATs are recruited to cytokine and growth factor receptor complexes upon their activation by ligand binding. STATs then homodimerize or heterodimerize, translocate to the nucleus and modulate transcription of target genes containing consensus DNA-recognition motifs called gamma activated sites. STAT proteins have been implicated in the regulation of muscle physiology and SC functions [29], [30]. DMD pathology has a significant inflammatory component, and immunological events are thought to play both reparative as well as injurious roles in the disease process [31]. However, a direct role for STAT proteins in the pathophysiology of DMD or other MDs has, to our knowledge, not been reported.

In the present study, we observed dynamic changes in S1P signaling after muscle injury. S1P deficiency due to disruption of Sphk1 impaired muscle regeneration and SC recruitment to injured fibers, as well as the proliferation and differentiation of SC-derived myoblasts *in vitro*. In contrast, S1P accumulation due to disruption of Sgpl1, which encodes SPL, enhanced SC-derived myoblast functions. Mdx mice that serve as a model of MD exhibited low circulating S1P levels consistent with evidence of enhanced S1P catabolism. Administration of a nontoxic SPL inhibitor blocked S1P degradation and improved mdx mouse SC activation and muscle regeneration. Our results suggest that an S1PR2/STAT3 signaling pathway leading to suppression of cell cycle inhibitors is responsible for S1P-mediated SC activation. Further, we show that SPL modulates SC functions *in vitro*, and pharmacological SPL inhibition *in vivo* enhances the recruitment of endogenous SCs into the cell cycle early in the muscle regenerative process, thereby improving muscle regeneration in a mouse model of MD.

## Results

### S1P synthesis, metabolism and signaling respond dynamically to muscle injury

**Figure 1:**
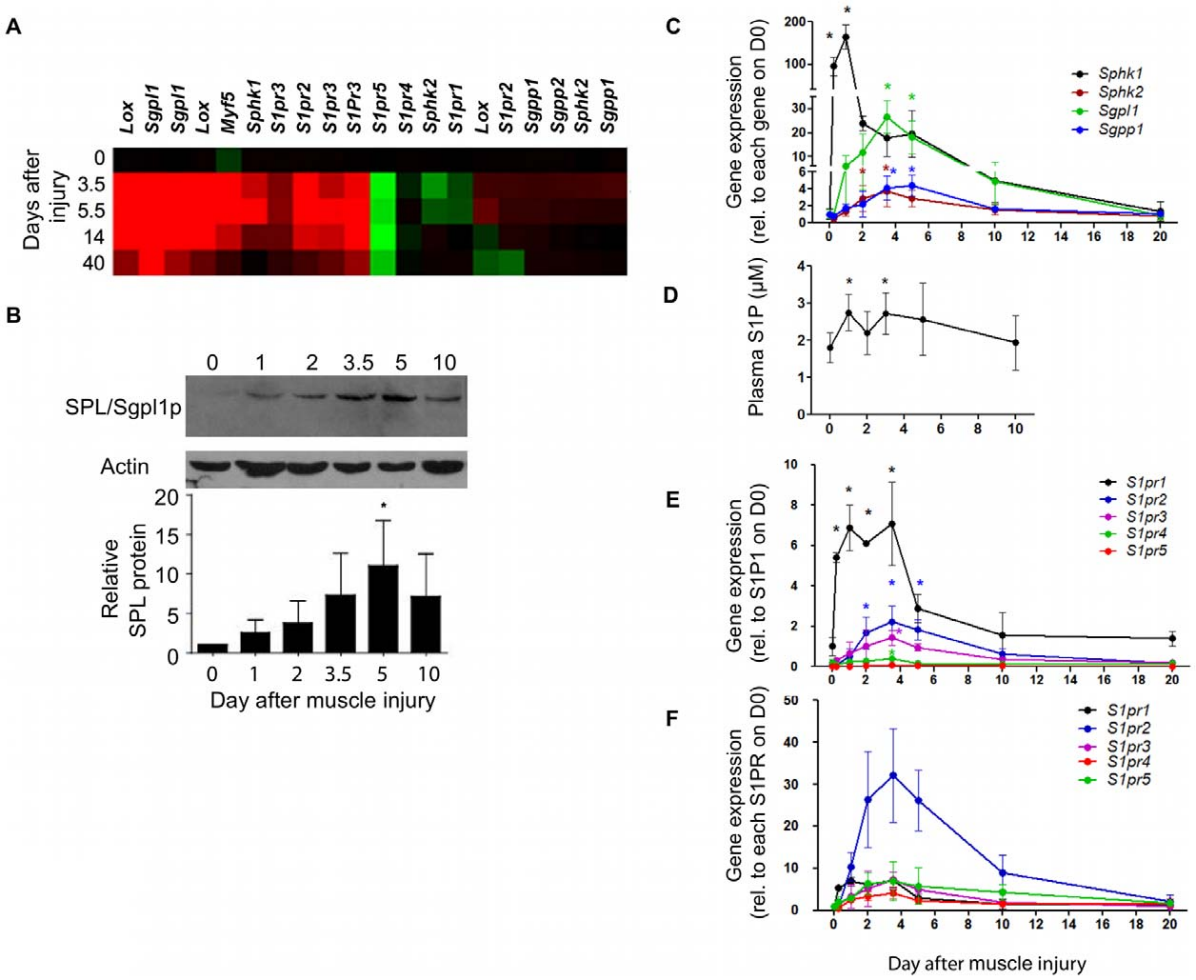
Dynamic changes in S1P signaling after muscle injury. A) Heat map of S1P receptors, S1P metabolic genes and other markers in WT C57BL/6 mouse skeletal muscle from day 0–40 after NTX injection. Red is induced, green is repressed relative to the mean. Note: two probes were available for some genes. *Lox* = lysyl oxidase; *S1pr1–5* = S1PRs; *Sgpl1* = SPL. *Sgpp* = S1P phosphatases; *Sphk* = sphingosine kinases. B) SPL protein (Sgpl1p) expression is induced after muscle injury, as determined by immunoblotting (above) and imageJ quantification relative to day 0 (uninjured muscle) and normalized to actin (below). C) Relative gene expression of *Sphk1, Sphk2, Sgpl1* and *Sgpp1* (normalized to *Gapdh*) were evaluated in whole muscle over time after injury by qRT-PCR. Gene levels are depicted relative to their own baseline levels (day 0 set as 1 for all genes). D) Corresponding plasma S1P levels were measured by LC-MS, E) Relative gene expression of S1PRs was determined as in “C” and is depicted relative to *S1pr1* (day 0 set as 1 for *S1pr1* only). F) S1PR induction is shown relative to baseline levels for each receptor (day 0 set as 1 for all genes). * indicates significant difference compared to day 0, p≤0.05. Data are means ± SD; n = 3–5/group.

S1P signaling has been implicated in various aspects of muscle biology [25]. However, the global effect of muscle injury on S1P signaling and metabolism has not previously been characterized *in vivo*. We previously performed temporal expression profiling on whole muscle tissue in the mouse notexin (NTX) muscle regeneration model in order to characterize myogenic genetic reprogramming after injury [32]. A search of this microarray database revealed that the expression levels of several genes involved in S1P metabolism and receptor signaling change over time after muscle injury (**Figure 1A**). The SPL gene (*Sgpl1*) was significantly upregulated on day 3.5, concomitant with transcriptional upregulation of the *Myf5* transcription factor, the ECM enzyme *lysyl oxidase* (*Lox*), the major SK *Sphk1*, and S1PR genes *s1pr2* and *s1pr3*. In contrast, *s1pr1*, *s1pr4*, *s1pr5*, and the gene encoding S1P phosphatase 1 (*Sgpp1*) were downregulated or unchanged at the time points interrogated in this array, and *Sphk2* expression results were inconsistent using two different probes. To confirm these findings, we first administered a single NTX intramuscular (i.m.) injection into the gastrocnemius muscles of C57BL/6 male mice (as described in Materials and Methods) and evaluated SPL gene and protein expression at different time points from day 0 (untreated) to day 10 after injury. Immunoblotting confirmed that muscle SPL protein expression increased over baseline levels by day 1 and reached maximal expression levels 5 days after injury (**Figure 1B**). To comprehensively characterize genetic changes affecting S1P metabolism and signaling in the aftermath of skeletal muscle injury, we administered a single NTX injection into the gastrocnemius muscles of C57BL/6 male mice as described above and followed the gene expression of S1PRs and major genes of S1P metabolism over time from 6 hours to 20 days in injured muscle by quantitative real time polymerase chain reaction (qRT-PCR). Within 6 hours after injury, we observed a 100-fold induction of *Sphk1*, followed by upregulation of *Sphk2*, *Sgpl1* and *Sgpp1* on days 3–5 or beyond after injury (**Figure 1C**). Measurement of S1P in the plasma of C57BL/6 mice under baseline conditions by liquid chromatography mass spectrometry (LC-MS) revealed circulating S1P levels of approximately 2 µM, consistent with our previous findings [33], [34]. In response to focal muscle injury, plasma S1P levels were found to increase by 50%, (**Figure 1D**), a perturbation that is known to exert physiological effects in other contexts [33]–[35]. We next characterized S1PR expression at baseline and in injured muscle. The *s1pr1* gene expression levels in whole muscle exceeded those of the other four S1PR subtypes at rest and after injury (**Figure 1E**). From 6 hours through day 3, *s1pr1* expression increased 5-fold and decreased thereafter, diminishing to near baseline levels by day 5. In contrast, a 25-fold increase in *s1pr2* expression was observed by day 3 and remained high through day 5 (**Figure 1F**). The expression levels of genes encoding S1PR3-5 also increased over time but remained low compared to those encoding S1PR1 and S1PR2 (Figures 1E and F). These findings establish that dynamic changes in S1P metabolism and signaling occur in the skeletal muscle fiber and microenvironment in response to injury.

### SphK1 is required for maximal muscle repair

**Figure 2:**
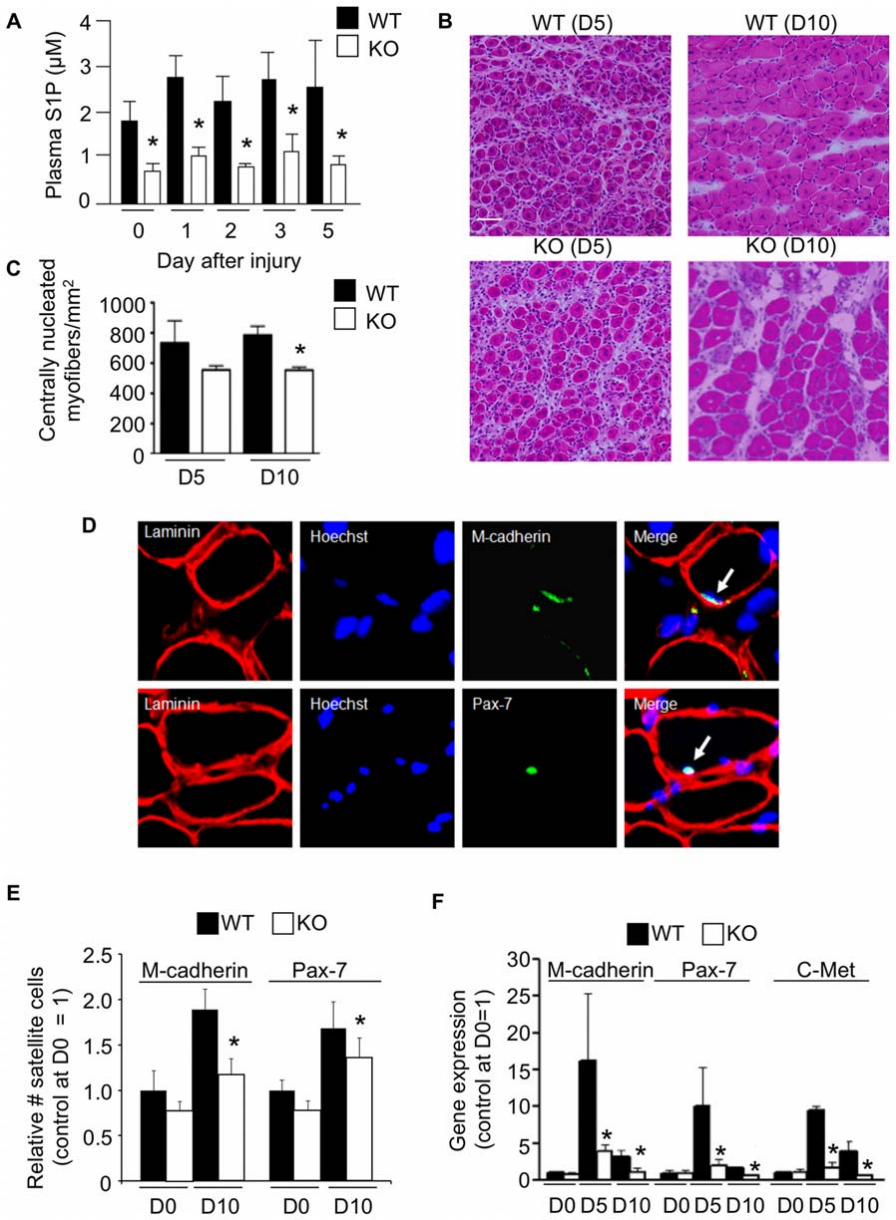
SphK1 disruption and S1P deficiency impairs muscle regeneration and SC recruitment. NTX injury was performed in WT C57BL/6 mice and SphK1 KO mice in the C57BL/6 background. Blood plasma and gastrocnemius muscles were harvested at different time points after injury. A) Plasma levels of WT and KO mice. B) Representative H&E stained frozen muscle sections of WT and SphK1 KO mouse muscles at the point of maximal injury. Scale bar = 50 microns. C) Fiber counts at days 5 and 10. D) Representative immunofluorescence staining on injured C57 BL/6 muscle sections with Hoechst (blue) for nucleus, laminin (red) for basal lamina and Pax-7 (green) or M-cadherin (green) for SCs. Arrow shows merged signals. E) Quantitation of SCs, identified by sublaminar mononucleated cells expressing Pax-7 or M-cadherin in WT and SphK1 KO muscles at baseline (day 0) and 10 days after injury. * p≤0.05 (KO at day 10 compared to WT at day 10 for both markers). F) Gene expression of SC markers (Pax-7, M-cadherin and c-Met) in uninjured (D0) and injured muscles at 5 and 10 days post injury. Data are expressed as means ± SD; n = 4 for immunostained cryosections; n = 3 for Western blot analysis; n = 3–5 for qRT-PCR; * indicates a significant difference between WT and KO at that time point, p≤0.05.

Use of nonspecific SK inhibitors has implicated a role for S1P in muscle regeneration [23]. We wished to confirm this finding using a genetic model system and to further establish whether SphK1 plays a specific role in muscle regeneration, as implicated by the profound *Sphk1* upregulation observed in injured muscle. Toward that end, we compared S1P levels and muscle regeneration capacity in WT mice versus SphK1 KO mice that lack the major enzyme required for S1P biosynthesis, but which are viable and healthy by virtue of an intact *Sphk2* gene [36]. S1P levels in SphK1 KO plasma were consistently between 40% to 57% of the circulating S1P levels observed in WT plasma at day 0 and through day 5 post-injury (**Figure 2A**). Muscle regeneration was compared in WT and SphK1 KO mice by examining the appearance of Hematoxylin & Eosin (H&E)-stained frozen sections and by quantification of nucleated, regenerating muscle fibers found at the point of maximal injury determined by serial sectioning and examination of the entire muscle, as described in Materials and Methods. As shown in Figure 2B, on day 5 after NTX injury both WT and SphK1 KO muscles contained fibers with centralized nuclei, indicating the presence of muscle regeneration at the point of maximal injury. At that time, there was no discernable difference between WT and SphK1 KO muscle fibers. However, by day 10 after injury, a compact assembly of centrally nucleated regenerating fibers was observed at the point of maximal injury in the WT muscles. In contrast, the regenerating fibers in the SphK1 KO muscles at day 10 appeared more sparsely organized, and quantification of regenerating fiber number/mm^2^ revealed a statistically significant reduction in regenerating fiber numbers in SphK1 KO muscles compared to WT muscles (**Figures 2B and C**).

**Figure 3:**
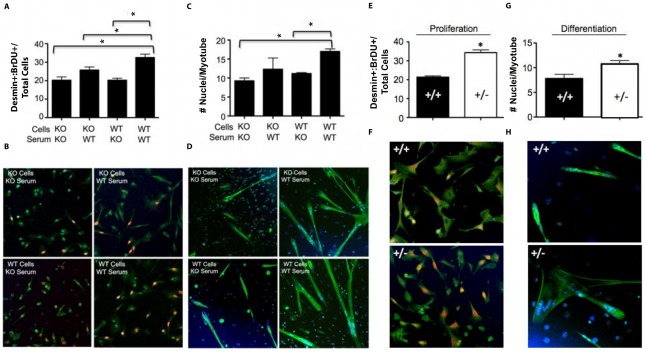
Function of SCs isolated from sphingolipid mutant mice. A) Proliferation of WT and SphK1 KO primary SCs isolated 3 days after NTX injury, measured by BrdU uptake of desmin-positive cells incubated with either SphK1 WT or KO serum. B) Representative immunofluorescence images for experiment in “A”. Desmin-stained cells, green; BrdU-stained nuclei, orange. C) Differentiation of SC-derived myoblasts as shown by myotube formation. D) Representative immunofluorescence images for experiment in “C”. Hoechst-stained nuclei, blue; eMHC-stained fibers, green. E) Proliferation of SPL heterozygous null (+/−) and littermate control (+/+) mouse primary SC isolated 3 days after injury. F) Representative images for experiment in “E”. Desmin-stained cells, green; BrdU-stained nuclei, orange. G) Differentiation of heterozygous null (+/−) and littermate control (+/+) mouse SC. H) Representative immunofluorescence images for experiment in “G”. Hoechst-stained nuclei, blue; eMHC-stained fibers, green. * indicates p≤0.05.

### SphK1 KO mice exhibit reduced SC activation and proliferation

To investigate whether inefficient muscle regeneration in SphK1 KO mice was associated with poor SC proliferation, resident SCs in tibialis anterior sections of WT and SphK1 KO mice isolated at rest and 10 days after injury were co-stained with fluorescent antibodies against the SC markers Pax-7 or M-cadherin, along with laminin to identify basal lamina and Hoechst staining of nuclei. SCs were identified as Pax-7 or M-cadherin positive, sub-laminar, mono-nucleated cells found in association with myofibers. A representative SC is shown in Figure 2D. Although SphK1 KO muscles appeared to harbor fewer SCs on day 0 compared to WT, the differences were not statistically significant. In contrast, 10 day post-injury SphK1 KO muscles harbored significantly fewer SCs than injured WT muscles, as determined by Pax-7 or M-cadherin staining (Figure 2E). Using qRT-PCR to measure the relative gene expression levels of SC markers c-Met, M-cadherin and Pax-7, we confirmed that SC markers were reduced in SphK1 KO muscles after injury (Figure 2F). Our results indicate that SphK1 is involved in mediating effective muscle regeneration and SC activation after injury.

S1P metabolism influences primary SC responses *in vitro*. Considering the importance of SphK1 in muscle regeneration and SC recruitment, we next examined the role of SphK1 in mediating SC functions in response to injury using a cell culture system. We wished to examine the intrinsic role of SphK1 in mediating SC functions in autocrine fashion, as well as the potential indirect contribution of SphK1 to regulating SC functions by influencing the muscle microenvironment. Toward that end, we isolated activated SCs from injured SphK1 KO and WT mice, cultured them in SphK1 WT or KO serum, and measured their ability to proliferate and differentiate. The combination of SphK1 KO cells and KO serum resulted in reduced proliferation compared to controls, as determined by BrdU uptake by desmin-positive cells (**Figures 3A and B**). A global (non-autonomous) requirement for SphK1 in mediating activated SC proliferation was identified, as shown by reduced proliferation of WT SCs in KO serum, whereas KO SC proliferation was increased in the presence of WT serum. Activated SCs also appear to have an intrinsic requirement for SphK1 to promote maximal proliferation, as WT serum did not restore KO SC proliferation to WT levels. Similar studies established that SCs also require SphK1 in non-autonomous fashion to support myotube formation, as determined by eMHC-positive myotubes after 48 hours in differentiation media (**Figures 3C and D**). SPL plays a major role in regulating S1P levels in cells and tissues. Mice homozygous for a gene-trap KO *Sgpl1* allele are unable to survive the neonatal period. In contrast, mice heterozygous for this allele exhibit a 30–40% increase in circulating S1P levels over WT levels and live normal lifespans [33]. In contrast to SCs derived from SphK1 KO mice, SCs isolated from SPL heterozygous null mice showed more robust proliferation (Figures 3E and F) and differentiation responses (Figures 3G and H) than littermate WT control SCs when incubated in the presence of their own serum. These studies establish that both biosynthetic and catabolic enzymes involved in S1P metabolism play an important role in the regulation of primary SC proliferation and differentiation.

### Mdx mice exist in a state of S1P-deficiency

**Figure 4:**
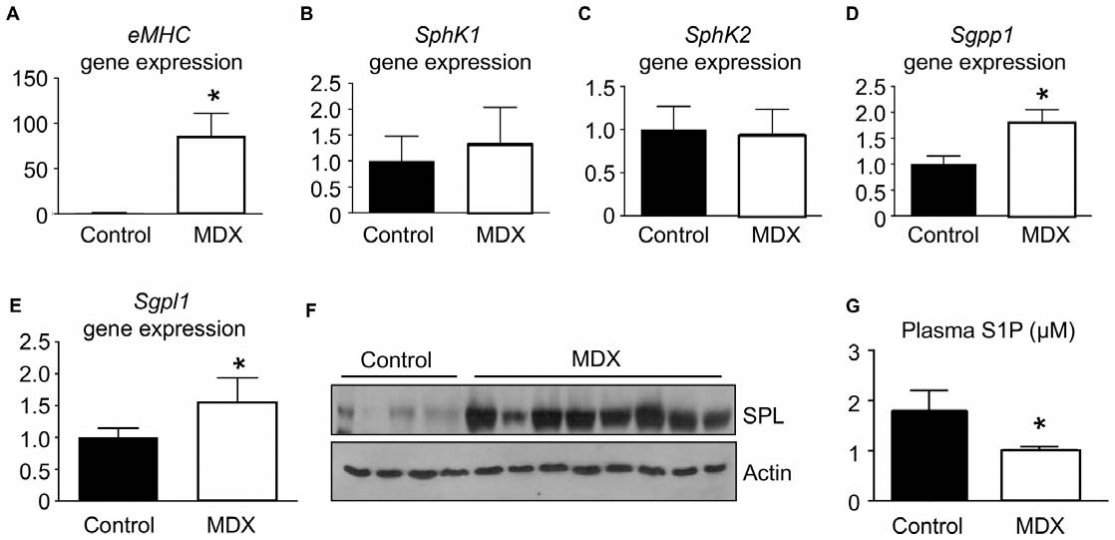
Dystrophic mice are characterized by an S1P-deficient state. Gene expression of eMHC, a marker of regenerating muscle, and key enzymes involved in S1P metabolism were examined by qRT-PCR in C57BL10/J and mdx (C57BL/10J background) mouse muscles under basal conditions. Data are means ± SD, n = 5/group; * indicates P<0.05. A) eMHC (*Myh3*) gene expression. B) *Sphk1* gene expression. C) *Sphk2* gene expression. D) *Sgpp1* gene expression. E) *Sgpl1* gene expression. F) SPL protein expression. G) Plasma S1P levels. Data are means ± SD, n = 4–5/group for plasma S1P; * indicates p≤0.05. Gene expression levels for qRT-PCR were normalized to GAPDH.

In consideration of our finding that S1P signaling is induced after muscle injury, we suspected that S1P signaling might also be altered in dystrophic muscles. To examine this possibility, we evaluated the levels of S1P and S1P-related gene expression in the dystrophic muscles of mdx mice, which harbor a point mutation in the murine dystrophin gene and are a rodent model for human DMD, in comparison to C57BL/10J control muscles [37]. Under basal conditions, eMHC mRNA was elevated in dystrophic muscles of mdx mice compared to control muscles, reflecting a state of continual degeneration/regeneration characteristic of dystrophic muscle (Figure 4A). No differences were observed between the two lines in gene expression levels of *Sphk1* and *Sphk2*, which suggests that S1P biosynthesis is not likely to be impaired in mdx mice (**Figures 4B and C**). In contrast, the gene expression of S1P catabolic enzymes *Sgpp1* and Sgpl1 were elevated compared to control muscles (Figures 4D and E). Consistent with this finding, SPL protein levels were elevated in mdx muscles compared to control muscles (Figure 4F). Importantly, circulating S1P levels of mdx mice were significantly lower than resting WT control plasma (**Figure 4G**). In fact, mdx S1P levels were similar to levels observed in SphK1 KO mice. These important findings reveal that dystrophic mdx mice exist in a state of S1P deficiency that is comparable to the S1P-deficient state of SphK1 KO mice and, thus, could have an impact on SC functions and muscle regeneration. However, unlike SphK1 KO mice, the mdx S1P deficiency appears to be due to enhanced S1P catabolism, rather than impaired biosynthesis.

### Inhibition of SPL in vivo enhances SC activation in mdx mice

**Figure 5:**
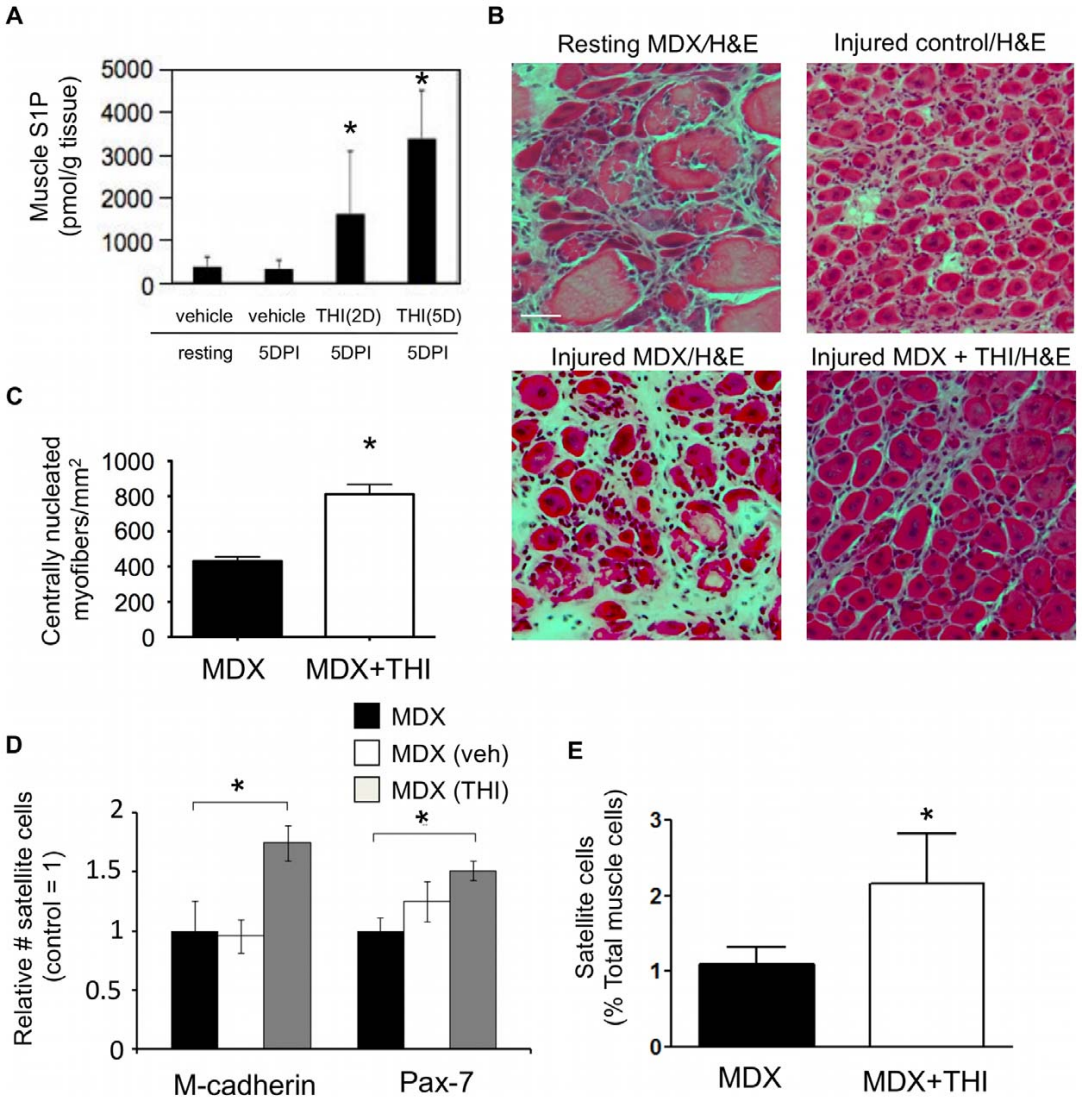
SPL inhibition improves muscle regeneration and SC recruitment in MD. A) Treatment of WT mice with THI for 2 days (2D) or 5 days (5D) results in elevated muscle S1P levels compared to vehicle-treated controls at 5 days post injury (5DPI); * indicates p≤0.05. Data are means ± SD, n = 4–5/group. B) Mdx mice received NTX+THI or vehicle daily on days −1 to 5. On day 5, mice were euthanized and gastrocnemius muscles were harvested. Ten µm tissue cryosections were obtained every 200 µm in sets of 4. H&E stained sections were examined for point of maximal injury. Representative photographs are shown. Scale bar = 50 microns. Recovering WT control and resting mdx muscles are shown for comparison. C) Fiber quantification of experiment in “B”. Centrally nucleated myofibers were counted in a field containing only regenerating fibers and normalized to 1 mm^2^ area. * p≤0.05. D) Quantification of SC *in situ* in mdx mouse muscles identified by M-cadherin or Pax-7 expression and localization beneath the basal lamina. * indicates p≤0.05. E) Flow cytometry analysis of freshly isolated muscle cells from mdx mice treated with THI or vehicle at 5 days post injury. SCs were identified as CD45-, CD31-, Sca1-, integrin alpha-7+ and CD34+ cells; * indicates p≤0.05, data are means ± SD, n  =  5/group.

We hypothesized that, like WT muscles injured with NTX, dystrophic muscles may also upregulate SPL in response to muscle injury. However, in the case of mdx mice, the injury results from intrinsic muscle fiber instability. We further hypothesized that the resulting chronically S1P-deficient state of dystrophic mice contributes to MD pathology by preventing efficient muscle repair. To address whether transient SPL inhibition could enhance muscle regeneration in mdx mice, we employed 2-acetyl-5-tetrahydroxybutylimidazole (THI), a nontoxic FDA-approved component of caramel food coloring that functions as an SPL inhibitor *in vivo* [38]. We have shown previously that THI treatment suppresses SPL activity and raises circulating plasma S1P levels by approximately 25–30% [33]. To assess the effect of SPL inhibition on muscle S1P levels, THI was administered in water for either 2 or 5 days to WT mice after NTX injury, and gastrocnemius muscle S1P levels were measured on day 5 post injury by LC-MS. THI treatment raised muscle S1P levels by 3–5-fold (**Figure 5A**). Mdx mice then received NTX injections in combination with orally administered THI or vehicle for 5 days after injury. Areas of uninjured mdx muscles showed the expected pathological findings including heterogeneous fiber size, the presence of large, necrotic fibers, small centrally nucleated fibers, and extensive inflammatory cellular infiltrates (Figure 5B). Five days after NTX injury, mdx muscles showed many areas lacking regenerating fibers at the point of maximal injury. In contrast, muscles from THI-treated mdx mice exhibited regenerating muscle architecture indistinguishable from regenerating WT muscle. THI-treated mdx muscles contained 2-fold more regenerating fibers than vehicle-treated mdx controls (Figure 5C). Quantification of SC numbers in regenerating mdx muscle using either M-cadherin or Pax-7 immunostaining and fluorescence microscopy demonstrated improvement in SC activation within the muscle fibers of THI-treated mdx mice compared to control mdx mice (Figure 5D). Flow cytometry approaches represent an unbiased method to identify SCs based on the presence or absence of specific cell surface markers. This approach was employed to confirm the effect of THI on SC proliferation that we had observed using histological methods as described above. Toward that end, CD45-negative, CD31-negative, Sca1-negative, alpha-integrin7-positive, CD34-positive SCs were quantified using flow cytometry of disaggregated muscle cells as described [39]. As shown in **Figure 5E**, THI treatment was associated with a significant increase in the number of SCs present in injured mdx muscle. These findings demonstrate that SPL inhibition enhances muscle regeneration and SC proliferation in a rodent model of MD.

### S1P recruits SCs through activation of S1PR2 and STAT3

**Figure 6:**
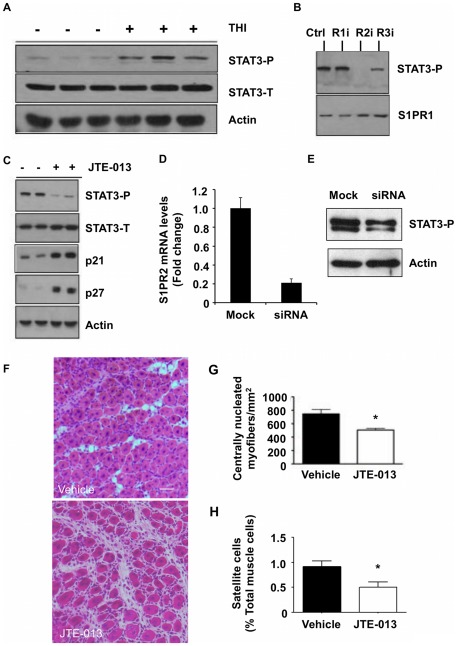
STAT3 and S1PR2 signaling interactions in muscle regeneration of WT and mdx mice. A) Mdx mice received NTX with or without THI treatment. Muscles were harvested on day 5 post-injury, and whole muscle extracts were examined for total STAT3 (STAT3-T) and phosphorylated STAT3 (STAT3-P) protein levels. Actin is used as a loading control. B) STAT3 activation in SC-derived myoblasts treated *in vitro* with 10 µM S1PR1 (R1i), S1PR2 (R2i), and S1PR3 (R3i) antagonists. S1PR1 is used as a loading control, as its expression is invariant under these conditions. C) WT SC-derived primary myoblasts were treated with S1PR2 antagonist (JTE-013) or vehicle, and whole cell extracts were examined for STAT3 phosphorylation, total STAT3 and p21 and p27 protein levels. Actin is used as a loading control. D) SC-derived primary myoblasts were treated with either 50 nM siRNA against S1PR2 or mock transfection. S1PR2 knockdown was confirmed by qRT-PCR. E) Whole cell extracts of control and S1PR2 knockdown cells were examined for phosphorylated STAT3 and actin protein levels by immunoblotting. p = 0.03 (siRNA vs. mock) for relative densitometric levels of STAT3-P/actin from two experiments performed in duplicates. F) WT mice were treated with NTX plus antagonist of S1PR2 (JTE-013) or vehicle by subcutaneous injection. Muscles were harvested 5 days post injury; regenerating fibers were imaged, and G) regenerating fibers were quantified. H) Flow cytometry analysis of SCs from WT mice treated with JTE-013 or vehicle at 5 days post injury; * indicates p≤0.05, data are means ± SD, n = 5/group.

S1P signaling has been implicated in SC activation [20], [26], [40]. Importantly, however, the molecular mechanism responsible for the contribution of S1P signaling to SC activation has not been determined. The STAT3 transcription factor promotes cell proliferation, survival and differentiation, has been implicated in mediating SC functions, and has been linked to S1P receptor signaling in cancer [28], [30], [41]–[46]. Therefore, we investigated the potential impact of THI treatment on STAT3 status in mdx muscles. STAT3 activation as shown by its phosphorylation on residue T705 was higher in injured muscles of THI-treated mdx mice compared to vehicle-treated mdx controls on day 5 post-injury (**Figure 6A**). To determine the S1PR requirements for STAT3 activation, we evaluated STAT3 phosphorylation in the presence and absence of S1PR antagonists *in vitro*. STAT3 phosphorylation was significantly reduced in SC-derived primary myoblasts treated with the S1PR2 antagonist JTE-013, whereas the S1PR1 antagonist W123 and the S1PR3 antagonist BML-241 had no impact on STAT3 phosphorylation (**Figure 6B**). STAT3 has been reported to negatively regulate cell cycle inhibitor proteins p21 and p27, thereby inducing cell cycle progression [41]. Importantly, SC-derived myoblasts treated with the S1PR2 antagonist JTE-013 showed a reduction in STAT3 phosphorylation, concomitant with an increase in p21 and p27 levels (**Figure 6C**). To further confirm these findings, we employed siRNA to knockdown the expression of S1PR2 and then evaluated the impact on STAT3 activation. As shown in **Figure 6D**, SC-derived myoblasts transfected with S1PR2 siRNA exhibited a 75% reduction in S1PR2 mRNA expression. S1PR2 knockdown resulted in a marked reduction in STAT3 phosphorylation, similar to the results obtained with JTE-013 (**Figure 6E**). Given these findings, we analyzed the effects of JTE-013 *in vivo* 5 days after NTX injury. We found that there was a significant decrease in centrally nucleated myofibers/mm^2^ after treatment with JTE-013 (**Figures 6F and G**). Further, JTE-013 treatment was associated with a significant decrease in the number of SCs when evaluated by FACS analysis (**Figure 6H**). These findings demonstrate that S1PR2 activation and downstream activation of STAT3 are essential for efficient muscle regeneration and SC proliferation.

In addition to SCs and SC-derived myoblasts, other cell types such as pericytes, endothelial cells and mesoangioblasts have been implicated in muscle regeneration and contribution to the SC niche [47]–[49]. To investigate the potential impact of S1P/S1PR2 signaling on STAT3 activation in these cell types, we applied exogenous S1P to representative lines of each, including human umbilical vein endothelial cells (HUVECs), the pericyte cell line C3H10T1/2 and the M25.2 clonally-derived populations of mesoangioblasts [50]. As shown in **Figure S1**, treatment of SC-derived myoblasts with 1 µM exogenous S1P resulted in robust activation of STAT3, which was prevented by pretreatment with 10 µM JTE-103. In contrast, HUVECs and M25.2 cells exhibited minimal STAT3 phosphorylation in response to S1P, and STAT3 phosphorylation was undetectable in C3H10T1/2 cells under the conditions we employed. However, high STAT3 activation was observed when C3H10T1/2 cells were treated with recombinant mouse Il-6 (data not shown).

### S1P activation of STAT3 is mediated via an S1PR2/Rho-GTPase-mediated pathway

**Figure 7:**
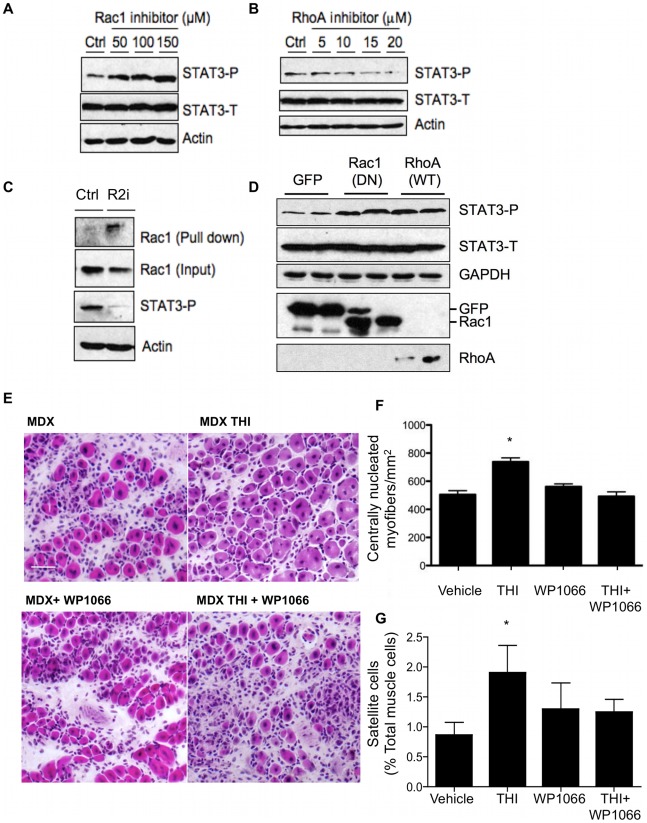
S1P activation of STAT3 is mediated via an S1PR2/RhoGTPase pathway. SC-derived myoblasts were treated with indicated dose of: A) Rac1 inhibitor, or B) RhoA inhibitor for 24 hours in complete media, and cell lysates were immunoblotted with indicated antibodies. Levels of total STAT3 (STAT3-T) and phosphorylated STAT3 (STAT3-P) were measured and compared to actin loading control. C) SC-derived myoblasts were treated with 10 µM S1PR2 antagonist JTE-013 (R2i) for 20 hours, cells were incubated in fresh media for 5 minutes with or without R2i, and activated Rac1 was pulled down with GST-PAK1 fusion protein and immunoblotted with Rac1 antibody. STAT3 activation (STAT3-P) also shown for this experiment. D) Myoblasts were transfected with GFP, dominant negative Rac1 (Rac1 DN) or wild type RhoA (RhoA WT) plasmid constructs. Forty-eight hours later, cell lysates were immunoblotted to detect total and phosphorylated STAT3, GFP, Rac1 and RhoA, with GAPDH used as a loading control. E) Representative micrographs (10×) of H&E stained 10 µm frozen sections taken from mdx gastrocnemius muscles 5 days post NTX-injury. Groups were treated with vehicle, THI, STAT3 inhibitor WP1066, or THI+WP1066. F) Quantification of regenerating myofibers/mm^2^ (n = 3/group). Scale bar = 50 microns. G) Analysis of SCs by flow cytometry as described in **Figure 5F**. Data are means ± SD, n = 5/group, * indicates p≤0.05.

We next sought to define the link between S1PR2 signaling and STAT3 activation. STAT3 activation is mediated by the ligation of various cytokines to gp130 and its associated receptors, which are then activated by JAK kinases, forming a docking site for STAT3 [28]. STAT signaling pathways are responsive to many intrinsic and environmental stimuli, including MAP kinases and Rho GTPases, known downstream targets of S1PR signaling [51]–[53]. We observed no effect of MAP kinase inhibitors on STAT3 phosphorylation in SC-derived primary myoblasts (data not shown). The Rho GTPase Rac1 has been shown to induce STAT3 activation in response to growth factors [54]. Surprisingly, SC-derived primary myoblasts treated with Rac1 inhibitors exhibited dose-dependent and significant STAT3 activation in comparison to vehicle-treated controls (Figure 7A). In contrast, RhoA inhibitors reduced myoblast STAT3 activation (**Figure 7B**). Importantly, when myoblasts were treated with an S1PR2 antagonist, Rac1 activation was enhanced, concomitant with STAT3 inactivation, as shown by reduced cellular levels of phosphorylated STAT3 (**Figure 7C**). Myoblasts were found to contain abundant Rac1 and low levels of RhoA and Cdc42 (data not shown). Therefore, to confirm the influence of Rac1 and RhoA on myoblast STAT3 activation, myoblasts were transfected with either a dominant negative construct of Rac1, a wild type RhoA construct or a GFP vector control, followed by measurement of Rac1, RhoA, total and phosphorylated STAT3 protein levels, with GAPDH serving as a loading control. As shown in **Figure 7D**, the expression in myoblasts of either dominant negative Rac1 or wild type RhoA resulted in higher activation of STAT3 in comparison to GFP control-transfected cells. These cumulative results indicate that in myoblasts, as in other cell types, S1P signaling through S1PR2 negatively regulates Rac1. However, in contrast to other cell types, inhibition of myoblast Rac1 releases STAT3 from suppression, thereby allowing it to repress cell cycle inhibitors and recruit SCs into the cell cycle.

### STAT3 activation is required to enhance muscle regeneration and SC functions through SPL inhibition in mdx mice

Importantly, treatment with the STAT3 signaling pathway inhibitor WP1066 reversed the positive effects of THI on mdx muscle recovery as well as SC proliferation after injury, demonstrating that THI potentiates SC proliferation through a STAT3-mediated pathway (**Figures 7E–G**). Our findings demonstrate a mechanistic link between S1P metabolism and S1PR2-dependent STAT3 signaling and suggest that this link is critical for THI-mediated improvement in muscle regeneration.

## Discussion

In this study, we have explored the function of the S1P signaling pathway within the context of muscle injury using the well-characterized NTX injury model. Initial exploratory studies characterized the impact of muscle injury on S1P signaling in WT murine muscle. Our findings demonstrate that, subsequent to acute muscle injury, dynamic changes occur in the expression of S1P biosynthetic and catabolic enzymes in whole muscle tissue. Marked Sphk1 induction was observed, followed by elevation of plasma S1P levels. Importantly, circulating S1P level elevations of the magnitude observed in our study have been implicated in mediating physiologically relevant effects such as radioprotection and ischemic cardioprotection [33]–[35]. Plasma S1P gradually returned to baseline levels concomitant with upregulation of SPL, the enzyme responsible for irreversible S1P degradation and the major regulator of S1P levels in cells and tissues. S1PR expression also changed over time after muscle injury, with S1PR1 and S1PR2 expression exhibiting the most significant changes.

To establish the physiological relevance of Sphk1 induction after muscle injury, we tested the efficiency of muscle regeneration in SphK1 KO mice. Our studies in this model demonstrate a specific requirement for SphK1 in both muscle regeneration and endogenous SC recruitment. Consistent with these findings, myoblast-derived SCs isolated from SphK1 KO mice exhibited a reduced capacity to proliferate and differentiate *in vitro*. Further, the S1P-deficient serum from SphK1 KO mice did not sustain optimal proliferation and differentiation of WT myoblast-derived SCs. These findings suggest that SphK1-derived S1P likely acts through a combination of autocrine mechanisms and effects on the muscle niche to promote SC functions and thereby facilitate regeneration after muscle injury.

We observed that SPL gene and protein expression rise after injury in WT mice and are also elevated under baseline conditions in the dystrophic muscles characteristic of mdx mice. Our results corroborate a previous study that found Sgpl1 upregulation as a component of a “chronic inflammatory response” pattern in mdx mice [55]. SPL is upregulated under a variety of stressful conditions including ischemia, infection, injury and DNA damage [56]. In contrast, the expression of enzymes required for S1P biosynthesis were not found to be elevated in mdx muscles. We propose that chronic muscle injury in MD may induce genetic changes that favor S1P catabolism and result in S1P deficiency, thereby impairing SC activation and muscle regeneration. The effect of chronic S1P depletion in dystrophic muscles, SCs and/or the muscle niche could in part explain the failure of genetic and cellular therapies to restore muscle health in MD.

Our findings in the mdx mouse model suggested the possibility that SPL inhibition could serve as a strategy to boost S1P levels, enhance endogenous SC functions and thereby promote muscle regeneration in MD. This notion is supported by our finding that myoblast-derived SCs isolated from SPL KO mice have greater capacity to proliferate and differentiate *in vitro* than SCs isolated from littermate controls. Genetic or pharmacological SPL suppression protects against other forms of injury including ischemia, DNA damage, and autoimmune and infection/inflammatory insults [33], [34], [57], [58]. Our results in mdx mice demonstrate that regeneration after injury of dystrophic muscles is improved by pharmacological SPL inhibition using the nontoxic food additive, THI. SPL inhibition by THI results in an expansion of endogenous SC pools, as determined by SC quantification using histological methods and analysis by flow cytometry. We speculate that the expansion of SCs with THI treatment results in a larger reserve population of SCs capable of activating with subsequent injury. It remains to be tested whether SPL inhibition using THI or other SPL inhibitors in recipients of cellular therapy will facilitate donor SC proliferation and incorporation into dystrophic muscles.

We have shown that STAT3 activation plays a critical role in mediating the positive effects of SPL-inhibition on muscle regeneration. This conclusion is supported by our findings that the muscles of THI-treated mdx mice exhibit enhanced STAT3 activation, whereas the increases in regenerating fiber numbers and SC recruitment after injury associated with THI treatment are attenuated by inhibition of STAT3-dependent signaling. Several reports have linked S1P signaling to the regulation of STAT3, including the recent study establishing that paracrine S1P signaling events are responsible for constitutive STAT3 signaling in cancer [46]. In addition, S1P and S1P-containing HDL activate STAT3 signaling in ventricular cardiomyocytes and in prostate cancer cells [59], [60]. While interleukin-6 and STAT3 are known to participate in muscle biology and SC functions [29], [30], the only published report we are aware of that links STAT3 to MD was an exploratory survey of transcription factors in mdx [61]. Whether other STAT3 activators could be useful in MD remains to be tested. It is interesting to note that the STAT3 activator LIF enhances muscle regeneration through the incorporation of transplanted donor myoblasts into injured and diseased muscle fibers [42], [62]–[64].

Our results have implicated a critical role for S1PR2 signaling in mediating SC recruitment and muscle regeneration. This notion is supported by our finding of reduced numbers of regenerating fibers and endogenous SCs in response to muscle injury in mice pretreated with the S1PR2 antagonist JTE-013. This effect occurs at least in part at the level of the myoblast, since activated STAT3 is markedly reduced in myoblasts treated with S1PR2 siRNA or with the S1PR2 inhibitor JTE-013 but not with other receptor inhibitors. Importantly, STAT3 has been shown to repress cell cycle inhibitor proteins including p21 and p27, which has been proposed as one of the major mechanisms responsible for STAT3's ability to promote cell proliferation [41]. We found that S1PR2 inhibition by JTE-013 in myoblasts reduces STAT3 activation and results in marked upregulation of p21 and p27. Our cumulative findings suggest that the activation of SCs by S1P may be mediated through a pathway involving activation of S1PR2 and STAT3, leading to the repression of cell cycle inhibitors and de-repression of the quiescent state.

Our results suggest that S1PR2 signaling activates STAT3 through a process that involves its reciprocal effects on small Rho GTPases. Specifically, we find that inhibition of S1PR2 in myoblasts correlates with Rac1 activation concomitant with STAT3 inhibition, whereas Rac1 inhibition or RhoA overexpression in myoblasts leads to activation of STAT3. The finding that S1PR2 activation leads to inhibition of Rac1 in myoblasts is consistent with the known functions of S1PR2 in other cell types [65], [66]. In contrast to the effect of S1PR2 on Rac1, RhoA has been shown to be activated by S1PR2 signaling [67], [68]. There is considerable evidence that wild type and mutant Rac1 and other Rho small GTPases can activate STAT3, especially within the context of cancer [53]. However, our findings suggest that in myoblasts inhibition of Rac1 and activation of RhoA by S1PR2 contribute to activation of STAT3.

STAT3 plays a role in rodent and human SC proliferation, migration and differentiation [30], [42]–[44], [69]. Therefore, we consider it likely that S1P signaling plays a critical role in multiple SC functions through its impact on STAT3 signaling. However, in our investigation of primary SCs from WT and sphingolipid mutant mice, it was not possible to segregate proliferation from differentiation effects, since a change in proliferation rate would reduce the number of SCs available to undergo fusion, nuclear accretion, and myotube formation. Therefore, additional studies will be required to specifically delineate the involvement of S1P/S1PR2/STAT3 signaling in SC differentiation and migration.

In summary, we have found that S1P signaling changes dynamically in response to muscle injury, plays a role in SC activation and muscle regeneration through an S1PR2/STAT3-dependent signaling pathway, and that SPL can be targeted to enhance muscle regeneration in a model of MD. Many of the known functions of S1P, including its role in angiogenesis, nitric oxide metabolism, innate and adaptive immunity, calcium homeostasis and cytokine and growth factor signaling, are important components of the physiological response to muscle injury. It will be important to determine how S1P-dependent signaling and SPL inhibitory strategies impact the many facets of muscle regeneration. Considering the recently described role of S1P as a histone deacetylase inhibitor, it is also possible that S1P influences epigenetic reprogramming in regenerating muscle [70]. The ubiquitous role of S1P signaling in physiology raises the concern that targeting SPL may be too toxic for practical use. However, humanized SPL mice exhibiting 10–20% of WT SPL activity levels live a normal lifespan, are healthy, reproductive and exhibit lymphopenia as their only phenotype [71]. Human clinical trials using small molecule inhibitors of SPL appear promising for the treatment of autoimmune diseases [58], [72]. These findings suggest that brief, intermittent or incomplete pharmacological SPL suppression is a feasible therapeutic strategy for enhancing SC functions in MD and other diseases of skeletal muscle. Future studies employing other murine models and large animal models of MD should help to clarify this issue.

## Materials and Methods

### Reagents

Antibodies to BrdU [BU1/75 (ICR1)], M-cadherin, SphK1 and S1PR1 were purchased from Abcam (Cambridge, MA, USA). Developmental eMHC antibody (clone RNMy2/9D2) was from Vector Laboratories (Burlingame, CA, USA). Antibodies to desmin (clone DE-U-10), laminin and actin were from Sigma-Aldrich (St. Louis, MO, USA). Hoechst nuclear stain was from VWR scientific (Radnor, PA, USA). Pax7 antibody was from Developmental Studies Hybridoma Bank (Iowa City, IA, USA). Antibody to murine SPL was generated as we described previously [73]. Antibodies to p21, p27 and GAPDH were from Santa Cruz Biotechnology, Inc. (Santa Cruz, CA, USA). Antibodies to total and phosphorylated STAT3 were from Cell Signaling Technology (Danvers, MA, USA). Antibody to Rac1 was from BD Transduction Laboratories (San Jose, CA, USA). Fluorophore-conjugated secondary antibodies (Alexa Fluor) were obtained from Molecular Probes (Eugene, OR, USA). Fluorophore-conjugated antibodies anti-CD31 PE-Cy7, anti-CD34 efluor 450, anti-CD45 FITC, anti-Sca-1 PerCP Cy5-5 were from eBioscience (San Diego, CA, USA). Anti-integrin alpha-7 APC was from R&D Systems (Minneapolis, MN, USA). Rho A inhibitor (H-1152), S1P and S1PR antagonist (BML-241, W123) were obtained from Cayman Chemical (Ann Arbor, MI, USA). S1PR2 antagonist, JTE-013 was obtained from Tocris Biosciences (Ellsville, MO, USA). STAT3 inhibitor, WP1066 and Rac1 inhibitor 1 were obtained from EMD Biosciences (Gibbstown, NJ, USA). NTX, Ponceau S, protease inhibitors, collagenase Type IIA and dispase were purchased from Sigma-Aldrich (St. Louis, MO, USA). Enhanced chemiluminescence (ECL) was from Amersham Bioscience (Piscataway, NJ, USA). SYBR-Green PCR master mix and reverse transcription kit were purchased from Applied Biosciences (Foster City, CA, USA). PCR primers were from Integrated DNA Technology (San Diego, CA, USA). DMEM, Ham's F-10, 20% FBS and horse serum were from Mediatech (Manassas, VA, USA). Penicillin-streptomycin was from Invitrogen (Carlsbad, CA, USA). FGF was from Millipore (Billerica, MA, USA). Opti-MEM was purchased from UCSF Cell Culture Facility (San Francisco, CA, USA). Recombinant mouse Il-6 (R&D systems, Minneapolis, MN, USA).

### Animal Subjects

Mice were housed under specific pathogen-free conditions. The animal protocol used in these experiments was approved by the Institutional Animal Care and Use Committee of the Children's Hospital Oakland Research Institute, and is in accordance with the National Institutes of Health guidelines for use of live animals. C57BL/6, C57BL10/J and mdx mice (C57BL/10ScSn-Dmdmdx/J) were obtained from Jackson Laboratories (Bar Harbor, ME, USA). Sphk1 KO mice [36] were described previously. SPL gene-trap KO and reporter mice [74] were from Philippe Soriano (Mount Sinai School of Medicine, NY, USA). Age- (2–3 months) and gender-matched controls were used in all procedures.

### Microarray Studies and Data Analysis

Analysis of temporal changes in S1P-related gene expression during the course of gastrocnemius muscle regeneration after NTX injury of WT mice was performed as described previously [32]. All subsequent studies using NTX injury were modified to avoid skin incision.

### Focal Muscle Injury and Isolation

Focal muscle injury and analysis of muscle regeneration *in vivo* was performed using single NTX injection (20 microliters) into tibialis anterior and gastrocnemius muscles. Direct intramuscular injection with NTX dissolved at 10 µg/mL in sterile PBS was used. Mice were euthanized by CO_2_ inhalation and cervical dislocation 6 hours to 20 days post-injury. Whole muscle (uninjured and regenerating) was isolated and prepared for (1) qRT-PCR, (2) immunoblotting, (3) muscle S1P measurement, (4) and flow cytometry. Plasma was also collected for S1P measurement and tissue was also used for cryosectioning and histology (described below).

### Real-Time Quantitative RT-PCR

For qRT-PCR studies, cDNA was generated from 1 µg RNA using a reverse transcription kit. Real-time PCR reaction was carried out on an ABI 7900HT thermocycler (Applied Biosystems, Carlsbad, CA, USA) coupled with SYBR Green technology using gene specific primers (shown below) and cDNA reaction mixture. All results shown represent gene expression that has been normalized to GAPDH levels.

### Primer Sequences

*Sphk1*

Forward Primer CGTGGACCTCGAGAGTGAGAA

Reverse Primer AGGCTTGCTAGGCGAAAGAAG

*Sphk2*

Forward Primer GCTTTCACCCATCGCTGAAG

Reverse Primer GGCAGGAACCCCGAAGAT

*Sgpl1*

Forward Primer GTTGGGCCGCCTTGATG

Reverse Primer GATGATCTGTTTGGTAGCTTCAACA

*Sgpp1*

Forward Primer CCCATTGGTGGACCTGATTG

Reverse Primer GATGAGCGGCGCATATTTG

*s1pr1*

Forward Primer ACCTAGCCCTCTCGGACCTATT

Reverse Primer CCCAGACAACAGCAGGTTAGC

*s1pr2*

Primer GCCATCGCCATCGAGAGA

Reverse Primer TGTCACTGCCGTAGAGCTTGA

*s1pr3*

Forward Primer GCCAGTCTTGGGAAATGACACT

Reverse Primer TGCCAGTTTCCCCACGTAA

*s1pr4*

Forward Primer CTGCCCGCCGCAAGT

Reverse Primer CACAAAGGCCACCAAGATCA

*s1pr5*

Forward Primer TGGCTGTGTGTGCCTTCATT

Reverse Primer GCGGACCAGCACCAAGAG

*Cdh15* (M-cadherin)

Forward Primer CCTGATGGGCAGTTCAAGATC

Reverse Primer CACGGACAGCACACCTTCAT

*Pax-7*

Forward Primer AAAAAACCCTTTCCCTTCCTACA

Reverse Primer AGCATGGGTAGATGGCACACT

*Met* (c-Met)

Forward Primer CAGCATCGCTCAAATTCAGAGA

Reverse Primer GGCCCAGCTGTTTCAGTGA

*Gapdh*

Forward Primer CCAGCCTCGTCCCGTAGA

Reverse Primer CGCCCAATACGGCCAAA

### Immunoblotting

Frozen muscles were homogenized in lysis buffer supplemented with protease inhibitors. Sodium orthovanadate and sodium fluoride (1 and 10 mM final concentrations, respectively) were also added to the lysates to inhibit phosphatase activity. Proteins were separated by SDS-PAGE and transferred by electroblotting to nitrocellulose membranes. Equivalent loading and transfer of proteins were verified by staining the blots with Ponceau S. Immunoblotting was performed using standard SDS-PAGE Western blotting as previously described in our laboratory [75]. Immunoreactive bands were detected by enhanced chemiluminescence and quantified by densitometric analysis of digitized autoradiograms with NIH Image 1.61 software.

### S1P Quantification

For S1P measurements, mouse muscle was homogenized in 0.5 ml of methanol using a glass homogenizer and a tip sonicator. Following homogenization, 1.0 ml of chloroform/methanol 1∶1 was added and the sample was incubated over night at 48°C. Sample was dried down and resuspended in 3 ml of chloroform/methanol 2∶1, and the extract was made basic by adding 50 µl of 1 M KOH in methanol. A two-phase separation was obtained by adding 0.5 ml of water. The aqueous phase was recovered and made acidic by adding 0.1 ml of concentrated acetic acid. Another two-phase separation was obtained by adding 1 ml of chloroform/methanol 2∶1 and the organic phase was recovered. S1P was extracted from 10 µl of mouse plasma by adding 0.4 ml of methanol followed by vortexing and incubation for 30 minutes at 30°C. The sample was spun in a tabletop centrifuge at 14,000 g and the supernatant was recovered. 17C-S1P was used as an internal standard. Lipids were separated on a C18 column (2.1×50 mm; Kinetex) (Phenomenex, Torrance, CA, USA) at a flow rate of 0.25 ml/min. The gradient used was from 45% to 99% methanol containing 1% acetic acid and 5 mM ammonium acetate. The data were acquired in positive mode on a Micromass Quattro LCZ MS (Waters Corp., Milford, MA, USA). Lipids were identified based on their specific precursor and product ion pair and quantitated using multiple reaction monitoring as described [76].

### SC Quantification by Flow Cytometry

The gastrocnemius muscles harvested after focal NTX injury were subjected to enzymatic dissociation for 2 hours. The cell suspension was filtered through a 70 µm cell strainer, followed by centrifugation and washing. One million cells were counted and incubated with fluorescently conjugated monoclonal antibodies for anti-CD31, anti-CD34, anti-CD45, anti-Sca-1 anti-integrin alpha-7 on ice for 45 minutes in the dark, followed by washing and centrifugation. Antibody/fluorophore combinations used were: anti-CD31 PE-Cy7, anti-CD34 efluor 450, anti-CD45 FITC, anti-Sca-1 PerCP Cy5-5, anti-integrin alpha-7 APC. Positive events and gates were determined by comparing fluorophore signal intensities between the unstained control and each single antibody/fluorophore control. Appropriate isotype controls were used. SCs were identified using CD45-, CD31-, Sca-1-, CD34+ and integrin alpha-7+ markers. Data were acquired with the BD LSRFortessa flow cytometer, and in each sample 100,000 events were recorded. Subsequent analysis and flow cytometry plots were generated using FlowJo v.7.2.5 (TreeStar, Inc., Ashland, OR, USA).

### Bulk Activation of Myofiber-Associated SCs

Most of the procedures involving SCs were performed as previously described [77], [78]. Tibialis anterior and gastrocnemius hindlimb muscles were injected with NTX through the skin with two to five injury sites made for each muscle (0.1 µg NTX delivered per site of injury), and muscles were harvested at various assay-dependent time points. SCs were then isolated from harvested muscle for *in vitro* assays.

### SC Isolation and SC Derived Myoblast Culture

SCs were isolated as described previously [77]. Briefly, whole muscle was initially digested at 37°C in DMEM, penicillin (100 U/ml)–streptomycin (100 µg/ml), and 300 U/ml Collagenase Type IIA. SC-associated myofibers then underwent several trituration, sedimentation and washing procedures before continued enzymatic digestion with 4 U/ml Collagenase Type IIA and 10 U/ml Dispase. Myogenic purity was confirmed by the generation of proliferating, fusion-competent myoblasts after 24 hours in growth medium (Ham's F-10 plus 20% FBS and 5 ng/ml bFGF), and de novo myotube formation after 48–72 hours in differentiation medium (DMEM +2% horse serum, penicillin-streptomycin) [79]. For cell culture and analysis, injury-activated or uninjured SCs were cultured on 1∶500 ECM/PBS-coated chamber slides (Nunc, Rochester, NY, USA). To assess the *in vitro* efficiency of myogenic repair, cells were cultured in Opti-MEM in the presence of specified sera for 24 hours (to examine proliferating myogenesis), or switched to differentiation medium for an additional 48–72 hours (to examine fusion competence). In some experiments, specific S1PR antagonists were added to culture (see Reagents for details). All transfections except siRNA (see below) were performed by Ca_2_PO_4_ precipitation method. Twenty-four hours later, the transfection mix was replaced with normal media (Ham's-F10 plus 20% FBS, and 5 ng/ml bFGF). All treatments were initiated 24 hours after the media change.

### Other Cell Culture Conditions

Pooled human umbilical vein endothelial cells (Lonza Inc, Allendale, NJ, USA) Lot # 0000115425 Cat# C2519A were propagated in Endothelial cell medium bullet kit-2 (Lonza Inc, Allendale, NJ, USA). C3H/10T1/2 Clone 8 pericytes Embryonic- Lot# 58613480 (ATCC, Manassas, VA, USA) were propagated in Eagle's Basal Medium with 2 mM L-glutamine, 1.5 g/L sodium bicarbonate and Earle's BSS (University of California San Francisco Cell Culture Facility, San Francisco, CA, USA) with 10% heat inactivated FBS (Invitrogen, Grand Island, NY, USA). M25.2 mesoangioblast cells were maintained in Iscove's medium supplemented with 10% FBS, 0.1 U/ml penicillin, 0.1 µg/ml streptomycin, 2.0 mM L-glut, 0.1 mM nonessential amino acids and minimal essential medium vitamin solution (Gibco, Carlsbad, CA, USA) and 10 ng/ml purified LIF (Chemicon International, Temecula, CA, USA).

### Histological Procedures

Most histological procedures were performed as previously described [77]. For *in vitro* studies, 10 µM BrdU was added for 2 hours prior to cell fixation (performed in 70% ethanol/PBS) and subsequent immunoassays. BrdU-specific immunostaining required nuclear permeabilization with treatment of 4N HCl. Following cell permeabilization in PBS containing 1% FBS and +0.25% Triton X-100, primary antibodies were incubated overnight at 4°C and followed by multiple washes in PBS containing 1% FBS. Fluorophore-conjugated, species-specific secondary antibodies (diluted 1∶500 in PBS containing 1% FBS) were added for 1–2 hours at room temperature. For histological assays, dissected muscle was treated in a 25% sucrose/PBS solution, embedded in OCT compound (Tissue Tek, Torrance, CA, USA) and frozen in liquid nitrogen-cooled isopentane. Ten-micron thick cryosections were cut using a Tissue-Tek Cryo3 Cryostat from Sakura (Torrance, CA, USA). Pax7 and M-cadherin SC quantification were performed as follows:

Tibialis anterior muscles received single, focal injury injections of NTX to muscle belly centers. At 10 days following injury, animals were euthanized (isoflurane), and injured/uninjured control muscles were harvested. Isolated muscles were incubated in ice-cold 25% sucrose/PBS solution for approximately 4 hours until sedimentation, followed by freezing in OCT compound. Cryosections (10 µm) were performed at 200 µm increments throughout the entire muscle, including the whole volume of injured tissue. Serial sections were then examined by hematoxylin and eosin staining to determine the site of injury “epicenters”. These sections were subsequently used for immunostaining and analysis of Pax7 and M-cadherin SC populations. SCs were initially identified based on localization of nuclear staining (Hoechst) within sub-laminar positions. Cell identity was then confirmed by Pax7 or M-cadherin expression. Total numbers were determined from multiple epicenter sections of individually-injured muscles, and based on quantifications performed within normalized surface areas for all samples analyzed. Data shown for Figure 2E depicts the increase in SCs, at post-injury day 10, relative to basal state muscles (D0 = uninjured).

### Immunofluorescence

Muscle sections were air-dried, fixed in 4% paraformaldehyde and washed in PBS containing 0.01% Tween 20 (PBST). Sections were blocked with PBST containing 5% goat serum and 0.3% Triton X-100. After washing, sections were incubated with anti-Pax7 and anti-laminin antibodies, respectively, followed by incubation with secondary Alexa fluor 488 goat anti-mouse IgG and Alexa Fluor 546 goat anti-rabbit IgG in PBST containing 5% goat serum. Sections were then counterstained with Hoechst after washing and mounted in Vectashield mounting medium (Vector Labs, Burlingame, CA, USA). Fluorescence was observed with a Carl Zeiss Axioskop 50 fluorescent microscope, and images were captured using Nikon DS-Ri1 camera.

### Inhibitor Studies In Vivo

JTE-013 was administered by subcutaneous injection at a dosage of 1 mg/kg daily starting 1 day prior to muscle injury. WP1066, which was dissolved in a 20∶80 mixture of DMSO∶PEG, was injected intraperitoneally at a dose of 20 mg/kg daily starting 1 day prior to injury. For THI treatment, animals were injured with a single NTX injection as described above and were given 50 mg/L THI in drinking water containing glucose ad libitum from 2 to 5 days after injury (muscle S1P study), or from −1 to 5 days after injury (mdx study). The control group received glucose water only. Animals were sacrificed 5 days post injury, and tissues were collected.

### S1PR2 Knockdown by siRNA

SC-derived primary myoblasts were plated in six-well plates in Ham's F10 medium containing 20% FBS and 5 ng/ml FGF. After 24 h, 50 nM S1PR2 siRNA smartpools (Dharmacon, Lafayette, CO, USA) were transfected with Dharmafect 1 (Dharmacon, Lafayette, CO, USA). Control cells were similarly treated with transfection reagent only. Twenty-four hours later, the transfection mix was replaced with Ham's-F10 plus 20% FBS, and 5 ng/ml bFGF. Forty-eight hours after transfection, the cells were evaluated for S1PR2 mRNA expression and phospho-STAT3 protein levels.

### Rac1 GTPase Activation Assay

Rac1 activation assays were performed as described previously [80]. Briefly, myoblasts were grown in 100 mm culture plates to 80% confluency. Cells were treated with S1PR2 antagonist JTE-013 for 6 hours. After washing, cells were lysed in cold lysis buffer containing 20 mM Tris-Cl, pH 7.4, 150 mM NaCl, 1% Triton X-100, 10 mM MgCl_2_, 0.2 mM phenylmethyl sulfonyl fluoride and protease inhibitor cocktail. Cell lysate was cleared by centrifugation at 12,000 g for 10 minutes, and cell lysate was incubated with GST-Pak1 agarose beads for 1 hour. After washing, samples were separated on 12% SDS-PAGE. Activated Rac1 was detected by immunoblotting using Rac1 monoclonal antibody.

### Plasmid Constructs

Plasmid encoding human SphK1 (pBabe-GFP-SK1) was described previously [34]. Plasmid encoding enhanced green fluorescent protein (GFP), pcDNA3-EGFP (Addgene plasmid 13031), dominant negative Rac1 pRK5-myc-Rac1-T17N (Addgene plasmid 12984) and WT Rho A construct pRK5-myc-RhoA-WT (Addgene plasmid 12962) were purchased from Addgene (Cambridge, MA, USA) and kindly provided by Douglas Golenbock (University of Massachusetts), Gary Bokoch (Scripps Research Institute), and Alan Hall (Memorial Sloan Kettering Institute), respectively.

### Statistical Analysis

Values are expressed as means ± SD. Student's unpaired t-test was used to compare two data sets. Comparisons among three groups were tested by one-way ANOVA using Prism GraphPad version 3.02 (GraphPad Software, San Diego, CA, USA). When the P value obtained from ANOVA was significant, Tukey's test was applied to test for differences among groups. Significance was considered to be P<0.05.

## Supporting Information

Figure S1 **STAT3 activation in myoblasts and vascular cells treated with S1P ± S1PR2 inhibitor.** SC-derived myoblasts, HUVECs, C3H/10T1/2 cells and M25.2 cells were starved overnight in serum-free media. After starvation, a subset of cells were pre-treated with 10 µM JTE-013 for 30 minutes. Cells were then treated with 1 µM S1P dispersed in PBS with 4 mg/ml of fatty acid free BSA with or without the addition of 10 µM JTE-013 for an additional 30 minutes. Control cells received an equal concentration of BSA in serum-free media. Cells were lysed in the presence of protease and phosphatase inhibitors and immunoblotted. A) Immunoblotting of whole cell lysates show relative levels of phosphorylated STAT3 (STAT3-P), total STAT3 (STAT3-T) and actin. B) STAT3-P/STAT3-T ratio determined by densitometry quantification using ImageJ software. BSA control is arbitrarily set at 1, except for C3H/10T1/2 cells in which STAT3-P was undetectable.
(TIFF)
