# Correction: Sphingosine-1-Phosphate Enhances Satellite Cell Activation in Dystrophic Muscles through a S1PR2/STAT3 Signaling Pathway

**DOI:** 10.1371/annotation/7e7ac57d-30ae-4e49-9138-e3bdbe3491d2

**Published:** 2012-06-05

**Authors:** Kenneth C. Loh, Weng-In Leong, Morgan E. Carlson, Babak Oskouian, Ashok Kumar, Henrik Fyrst, Meng Zhang, Richard L. Proia, Eric P. Hoffman, Julie D. Saba

There was an error in the published Acknowledgments statement. The correct Acknowledgments are:

We would like to acknowledge Nelle Cronen for expert administrative assistance and Max Soghikian for technical support of immunoblotting studies. We would like to thank Suzanne Berry-Miller (University of Illinois) for her generous gift of the M25.2 mesoangioblast cell line. We dedicate this study to Dr. William Fishbein for his inspiration and guidance. 

