# Supplementary figures and images for: Sphingosine-1-Phosphate Enhances Satellite Cell Activation in Dystrophic Muscles through a S1PR2/STAT3 Signaling Pathway
Source: PLoS One. 2012 May 14;7(5):e37218. doi: 10.1371/journal.pone.0037218 (PMC3351440; doi:10.1371/journal.pone.0037218)

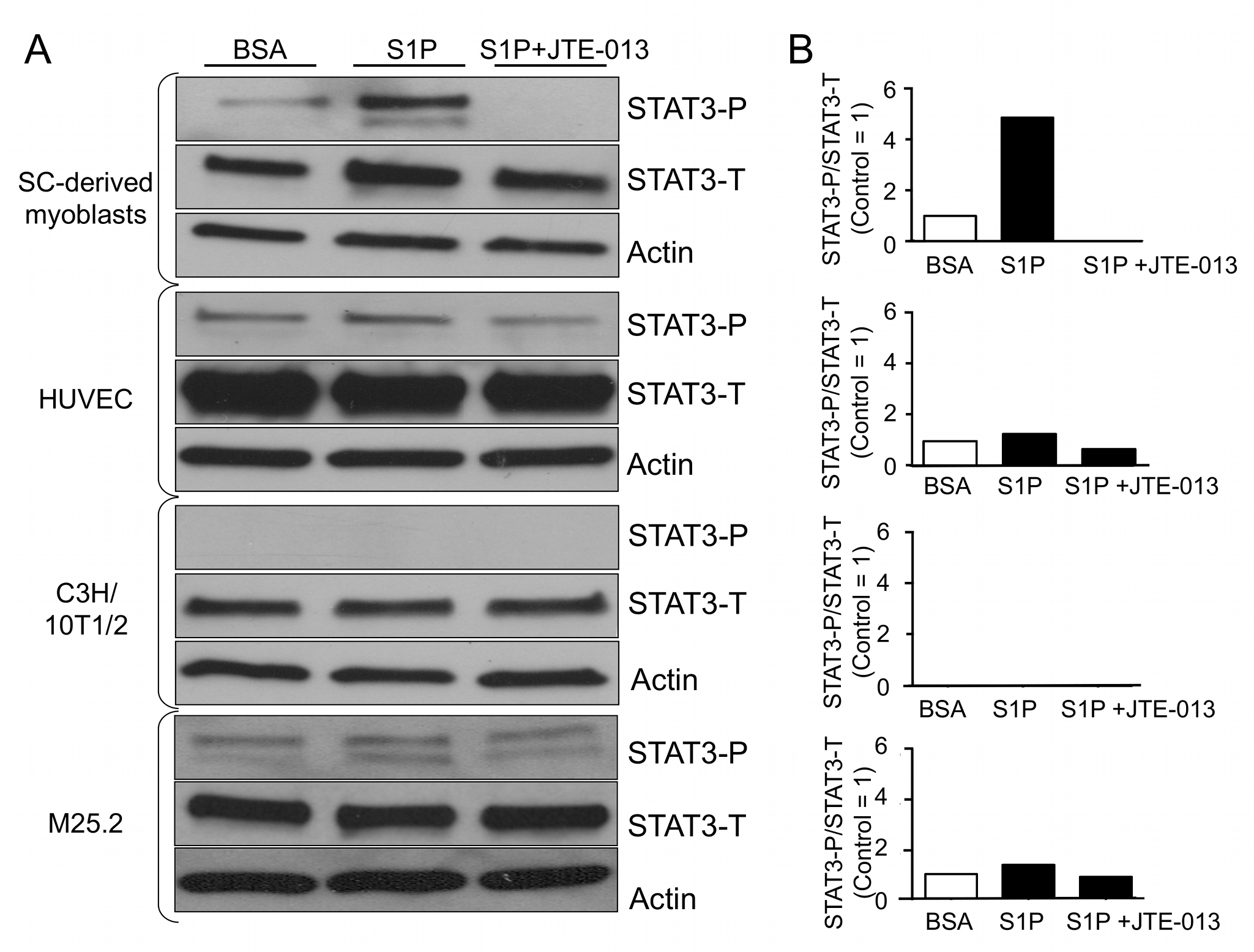

Supplement: Figure S1 — STAT3 activation in myoblasts and vascular cells treated with S1P ± S1PR2 inhibitor. SC-derived myoblasts, HUVECs, C3H/10T1/2 cells and M25.2 cells were starved overnight in serum-free media. After starvation, a subset of cells were pre-treated with 10 µM JTE-013 for 30 minutes. Cells were then treated with 1 µM S1P dispersed in PBS with 4 mg/ml of fatty acid free BSA with or without the addition of 10 µM JTE-013 for an additional 30 minutes. Control cells received an equal concentration of BSA in serum-free media. Cells were lysed in the presence of protease and phosphatase inhibitors and immunoblotted. A) Immunoblotting of whole cell lysates show relative levels of phosphorylated STAT3 (STAT3-P), total STAT3 (STAT3-T) and actin. B) STAT3-P/STAT3-T ratio determined by densitometry quantification using ImageJ software. BSA control is arbitrarily set at 1, except for C3H/10T1/2 cells in which STAT3-P was undetectable. (TIFF) [file pone.0037218.s001.tiff]
